# A resurrection study reveals rapid adaptive evolution within populations of an invasive plant

**DOI:** 10.1111/j.1752-4571.2012.00287.x

**Published:** 2012-09-09

**Authors:** Sonia E Sultan, Tim Horgan-Kobelski, Lauren M Nichols, Charlotte E Riggs, Ryan K Waples

**Affiliations:** 1 Biology Department, Wesleyan UniversityMiddletown, CT, USA

**Keywords:** contemporary evolution, introduced species, invasion dynamics, invasive plants, phenotypic plasticity, *Polygonum cespitosum*, rapid evolution, resurrection experiment

## Abstract

The future spread and impact of an introduced species will depend on how it adapts to the abiotic and biotic conditions encountered in its new range, so the potential for rapid evolution subsequent to species introduction is a critical, evolutionary dimension of invasion biology. Using a resurrection approach, we provide a direct test for change over time within populations in a species' introduced range, in the Asian shade annual *Polygonum cespitosum*. We document, over an 11-year period, the evolution of increased reproductive output as well as greater physiological and root-allocational plasticity in response to the more open, sunny conditions found in the North American range in which the species has become invasive. These findings show that extremely rapid adaptive modifications to ecologically-important traits and plastic expression patterns can evolve subsequent to a species' introduction, within populations established in its introduced range. This study is one of the first to directly document evolutionary change in adaptive plasticity. Such rapid evolutionary changes can facilitate the spread of introduced species into novel habitats and hence contribute to their invasive success in a new range. The data also reveal how evolutionary trajectories can differ among populations in ways that can influence invasion dynamics.

## Introduction

Evolutionary change is now increasingly recognized to occur on a timescale of decades or even years in natural populations of many taxa (Cody and Overton 1996; Hairston et al. 1999; Hartley et al. 2006; Franks et al. 2007; reviewed in Reznick and Ghalambor 2001; Parmesan 2006), often in response to recently imposed environmental challenges such as global climate change (Réale et al. 2003; Bradshaw and Holzapfel 2006), chemical pollutants (Antonovics et al. 1971), size-selective harvest (Olsen et al. 2004), and introductions of species to new geographic regions (Blossey and Nötzold 1995; Maron et al. 2004; Phillips and Shine 2004; reviewed in Mooney and Cleland 2001; Bossdorf et al. 2005; Sax et al. 2007). The potential for introduced species to rapidly evolve adaptations to newly encountered habitats can contribute critically to their success and spread as invasives. Hence, studies testing for rapid adaptive change in populations of introduced species add a crucial evolutionary dimension to the urgent practical issue of biological invasions.

A powerful approach for studying recent and rapid – i.e., ‘contemporary’ – evolution (Hendry and Kinnison 1999) is the use of resurrection protocols (Franks et al. 2007, 2008), in which genotypes are systematically collected from the same populations at different points in time and then raised under common conditions, to quantify evolutionary change within those populations over the collection interval. This approach has been used to document rapid evolution in bacteria (Lenski et al. 1991), aquatic invertebrates (Hairston et al. 1999), and annual plants (Davison and Reiling 1995; Franks et al. 2007), all of which can be stored as dormant propagules to allow for robust comparison of temporal samples. A resurrection approach makes it possible to answer several key questions about evolution in invasive non-native species. First, it can directly test whether evolutionary change has occurred within the introduced range. Such direct tests are a powerful addition to studies that infer evolutionary change by comparing geographically distant or native versus introduced populations of invasive taxa (e.g. Blossey and Nötzold 1995; Blair and Wolfe 2004; Maron et al. 2004; Lavergne and Molofsky 2007; Keller et al. 2009; reviewed in Bossdorf et al. 2005; Matesanz et al. 2010). Second, a resurrection approach can assess evolutionary adaptation as a factor in the invasion process by testing whether or not populations have evolved increased functional performance and reproductive fitness in the environments found in the introduced range. If designed appropriately, resurrection experiments can also reveal evolutionary change in trait plasticity expressed by individual genotypes, an aspect of adaptation that can contribute to an invasive species' spread across diverse and/or variable habitats (Sultan 2004, 2007). Finally, resurrection studies that compare patterns of change in multiple populations in an introduced range can inform predictions regarding the dynamics of a species' spread.

We used a resurrection protocol to test for recent adaptive evolution in established North American populations of *Polygonum cespitosum* (Bl.), an obligately annual plant native to temperate and subtropical Asia that was unintentionally introduced in the early 1900's (Paterson 2000; Kim et al. 2008). In its native range, *P. cespitosum* colonizes moist, shaded habitats such as stream banks and forest paths (Anjen et al. 2003). In northeastern North America, *P. cespitosum* was historically restricted to similar habitats (Sultan et al. 1998b), but in the past 10–15 years it has begun to spread into open sites that include microsites with high light intensity and potential moisture deficits as well as wet, high-light microsites (Fig. S1). Coincident with its increased ecological breadth, the species is now considered to be invasive in this part of its introduced range (Merhoff et al. 2003). In addition to the importance of *P. cespitosum* as an invasive plant in Northeastern North America, the species serves as an excellent model system for an introduced taxon in the process of making the transition to invasive spread.

We conducted a series of resurrection experiments on genotypes sampled from three New England *P. cespitosum* populations both before (1994) and during (2005) the species' ecological expansion into open sites (an interval of 11 generations). We measured ecophysiologically important traits as well as reproductive output to assess the adaptive value of any evolved changes. First, we compared functional and fitness responses of 1994-collected vs. 2005-collected genotypes to contrasting light and moisture levels, in two separate, single-factor glasshouse experiments. In a third experiment, we then compared responses of these genotypes to contrasting glasshouse ‘habitat’ treatments designed to simulate (a) the shaded, moist habitat initially colonized by *P. cespitosum* in northeastern North America, and (b) the full-sun, potentially drier conditions into which the species is now spreading. This suite of experiments documents remarkably rapid adaptive evolution of ecologically important traits and their plasticity in response to increased light intensity and moisture stress in this newly invasive introduced plant. More generally, the results demonstrate that altered patterns of adaptive trait expression can evolve in plant populations in surprisingly few generations.

## Materials and methods

### Experimental sample

The sample was drawn from three well-established *P. cespitosum* populations chosen to represent the range of shade habitats in which this species has historically occurred in this region (ARL = Arch Road, Leeds, MA; ORD = Katherine Ordway Preserve, Weston, CT; WEI = Weir Farm, Wilton, CT; site details in Sultan et al. 1998b). Achenes (1-seeded fruits) were collected from 10 to 12 plants ≥ 1.0 m apart in each population (September 1994, September 2005). Conditions at these sites have remained generally unchanged since 1994 (e.g. available photosynthetically active radiation (PAR) at *Polygonum* canopy height at ORD and WEI ranged from 3%–97% of full sun in 1994 and from 2%–100% in 2009 (data unavailable for ARL); T. Horgan-Kobelski, S. Matesanz, & S. E. Sultan, unpublished manuscript.

Field-collected achenes were germinated, raised to maturity, and allowed to produce self-fertilized achenes under uniform glasshouse conditions, to yield 64 inbred (selfed full-sib) lines (8–12 lines per population × 3 populations × 2 collection years). Plants were grown in 2:5 coarse sand: Pro-mix™ (Premier Horticulture Inc., Quakerstown, PA USA) fertilized with 20:20:20 soluble NPK (JR Peter's, Allentown, PA USA), with supplemental HID light when insolation between 0800 and 1800 h was < 750 μE, from November–February 2001 (1994 collections) or November–February 2006 (2005 collections). Achenes produced were air-dried and stored with desiccant at 4°C. Appreciable seed aging effects are unlikely under such conditions (Fenner and Thompson 2005); high germination rates for both inbreeding generations (average germination percentages ≥ 70%) confirm their absence. Still, since 1994 collections were stored longer than those from 2005, we tested for possibly confounding seed age effects. We experimentally tested for effects of achene age differences on seedling growth, the aspect of development most likely affected by seed age (Bewley and Black 1994; Smith and Berjak 1995). There was no difference (anova *P* > 0.5, *n* = 45) between 1994- and 2005- collected achenes in the biomass of d 10 seedlings raised in Open/Mesic glasshouse conditions (July 2007). We repeated this test after a second ‘refresher’ generation to compare the same 1994- and 2005-collected lines using achenes they produced in August 2006 (i.e., of identical age). In this second test, anova also showed no effect of collection year on seedling biomass (*P* > 0.2, *n* = 55). The identical growth responses of 1994 and 2005 achenes for certain traits and treatments (Results) are consistent with specific evolved changes rather than overall loss of vigor due to seed age. A field experiment using 1994- and 2005-collected achenes of identical age confirmed significant differences between collection years in physiological and phenological responses to full sun (T. Horgan-Kobelski and S. E. Sultan, unpublished manuscript).

For each of the three experiments, achenes from each inbred line were stratified in distilled H_2_O at 4°C for ≥ 4 weeks, sown into moist medium-grade vermiculite, and germinated on open glasshouse benches at 24°C day/20°C night. Either 1 (Moisture Experiment, Habitat Experiment) or two (Light Experiment) replicate seedlings per line were assigned to each growth treatment in a randomized complete block design.

## Experimental treatments

### Light experiment

Plants were grown in two glasshouse treatments, High vs. Low light (both in moist soil). Seedlings at the first true leaf stage were transplanted into 0.8L clay pots filled with 1:1:1 coarse sand: sterilized topsoil: Turface fritted clay (Profile, Buffalo Grove, IL USA) and 2.5 g per pot granular 15:8:12 NPK fertilizer (Agway, Syracuse, NY USA). Plants in High light received full sun (mean midday PAR c.1100 μmol/m^2^/s Red: Far-red ratio ≥ 1.07). Low-light plants were grown under adjustable metal frames covered with neutral 85% shade cloth (Hummert, Earth City, MO, USA) overlaid with green plastic filter strips (#138, Lee Filters, Burbank, CA USA) to simulate canopy shade (mean midday PAR c.160 μmol/m^2^/s; R:FR ratio 0.68–0.89 based on mid-day measurements with an SKR 110 R:FR meter (Skye Instruments, Llandrindod Wells, UK). Automatic systems delivered reverse-osmosis filtered H_2_O to pots via individual tubes (Chapin Watermatics, Watertown, NY, USA), supplemented with hand watering as needed. Mean soil moisture was maintained at 84–90% of field capacity in both Low and High light. Plants were grown in treatment March 31 to April 28, 2006 (replicate 1) or June 6, 2006 (replicate 2).

### Moisture experiment

Plants were grown in two glasshouse treatments, Dry vs. Moist soil (both in high light). Seedlings at the first true leaf stage were transplanted into filled pots as described above. Plants received full sun (mean midday PAR c.1100 μmol/m^2^/s Red: Far-red ratio ≥ 1.07). Mean soil moisture was maintained at 50% (Dry treatment) and 100% (Moist treatment) of field capacity by means of automatic watering systems supplemented with hand watering (see above). Plants were grown in treatment from June 30 to August 22, 2006.

### Habitat experiment

Plants were grown in two glasshouse treatments: Open/Mesic (full sun, 75% field capacity soil moisture) vs. Shade/Moist (85% simulated shade, field capacity soil moisture); details as above. These treatments mimic current habitats in *P. cespitosum*'s introduced range: in typical open sites, maximum light exceeds 90% of PAR and mean soil moisture is 47–80% of field capacity; in shaded sites, mean available light is 5–18% of PAR and mean soil moisture is 58–93% of field capacity (data in T. Horgan-Kobelski, S. Matesanz, & S. E. Sultan, unpublished manuscript).

## Data collection

### Fitness components

Lifetime total achene mass was calculated as air-dried mass of early-maturing achenes plus all mature/immature achenes, flowers, and reproductive support collected at final harvest (mature achenes comprise > 96% of this mass; S. E. Sultan, unpublished data). Early-maturing achenes were collected by rubbing inflorescences to release mature achenes yet allow other flowers to reach maturity, except in the Light Experiment, where they were collected by removing entire inflorescences that contained mature achenes. Mean individual achene mass was calculated from a random sample of 20 mature achenes per experimental plant, and total achene number estimated as (total achene mass/mean individual achene mass). These reproductive tissues were harvested after 9.5 weeks (Light Experiment repl. 2), 8 weeks (Moisture Experiment), and 10.5 weeks (Habitat experiment) in treatment.

### Allocation and morphology

After 4 weeks (Light Experiment repl. 1), 8 weeks (Moisture Experiment), or 10.5 weeks (Habitat experiment) in treatment, leaf and stem tissues of each plant were harvested, oven-dried (at 100°C for 1 h then 65°C for ≥ 48 h), and weighed to determine leaf and stem biomass. Three leaves from one primary branch per plant were scanned on an LI-3100 leaf area meter (Licor, Inc., Lincoln, NE USA), oven-dried, and weighed to determine specific leaf area (SLA; cm^2^/g). Whole-plant total estimated leaf area (TELA) was calculated as (SLA × leaf biomass). Intact root systems were stored at 4°C before being manually washed, oven-dried (at 65°C for ≥ 48h), and weighed to determine root biomass and the whole-plant ratio of root mass/leaf area (g root mass/m^2^ TELA) was calculated. Total plant biomass was calculated as (total achene mass + leaf mass + stem mass + root mass). Reproductive allocation was calculated as [(total achene mass/total plant biomass) × 100]. Root length was measured on a Comair Root Length Scanner (Hawker de Havilland, Melbourne, Australia) for a random sample of two entire root systems per population, collection year, and moisture treatment. These root systems were oven-dried at 65°C and weighed to determine specific root length (SRL; m root/g root mass). Allocation and/or fitness data were excluded from 4 (Habitat Experiment), 8 (Moisture Experiment), and 18 (Light Experiment) replicates due to harvest error or abnormal growth (3%, 6%, and 7% of experimental plants, respectively; results were not qualitatively affected by these exclusions).

### Physiological performance

*In situ* measurements were made on mature plants from a random subset of 5–9 lines per population from each collection year, between 1000–1500 h on sunny days from May 22–30, 2006 (Light Experiment) and August 5–14, 2006 (Moisture Experiment). Instantaneous photosynthetic rate (Photosynthesis; μmol CO_2_ m^2^/s) and stomatal conductance (Conductance; mol H_2_O m^2^/s) were measured on the most recent fully-expanded leaf from a 1° branch using a LI-COR 6200 photosynthesis system with LI-6250 gas analyzer (Licor, Inc.) and Q-Beam 1205 LED lamp (Quantum Devices, Inc., Barneveld, WI, USA). Each datum was the mean of three consecutive measurements during which chamber relative humidity changed < 1%. Data were excluded from 7 Light and 1 Moisture Experiment plants that were senescent. Leaf area tracings for rate calculations were measured with an LI-3100 leaf area meter (Licor, Inc.). Instantaneous water use efficiency (WUE; μmol CO_2_/mol H_2_O) was calculated as (Photosynthesis/Conductance).

## Data analysis

For each experiment, anovas using type III sums of squares (JMP version 7.0.1, SAS Institute, Cary, NC, USA) were performed to test for Treatment, Collection Year, Population, and interaction effects on each trait (Full **anova**). Air temperature and measurement day were included in Light Experiment physiology anovas. *Population* was treated as fixed because the three populations were chosen to represent the 1994 range of *P. cespitosum* habitats rather than as a random sample (see Sultan et al. 1998b). Populations within collection year were pooled in the SRL anova. Total achene mass and number and total plant biomass were Box-Cox transformed to meet anova assumptions (Zar 1999); other traits did not require transformation. *Post-hoc* Tukeys HSD tests were performed where direct comparisons among populations within a single treatment were of interest (Zar 1999). When population main effect or interaction terms were significant, separate anovas were performed to test for Treatment, Collection Year, and interaction effects within each population (Single-population anova). To investigate evolved differences in detail, one-way anova were performed testing for the effect of Collection Year within each population in each treatment (one-way anova). Because of very low statistical power in the SRL anova (*N* = 6 per collection year within each treatment), we use a significance threshold of ≤ 0.10 rather than the conventional 0.05 in this case (Filion et al. 2000; Heschel et al. 2004). Fitness data for the WEI population were removed from the Light Experiment dataset because the early-achene collection protocol used only in this experiment (see Data Collection, *Fitness components*) caused a disproportionate reduction in the total reproductive output of 2005 WEI genotypes, due to their delayed flowering in full-sun conditions (L. M. Nichols, unpublished data).

**Figure 1:**
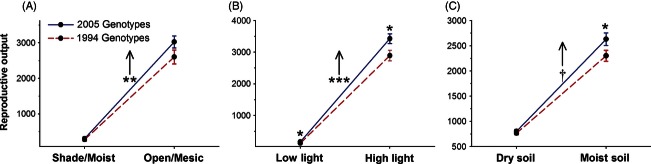
Rapid evolution of reproductive output (total achene number). Means are shown ± 1 SE for 26–29 genotypes per collection year pooled from three field populations. Vertical arrows indicate significant main effect of Year in full anova (see Table 1). Asterisks indicate significant effect of Year within each treatment (see *Data analysis*). ***P < 0.001; **P < 0.01; *P < 0.05; †*P* ≤ 0.10.

**Figure 2:**
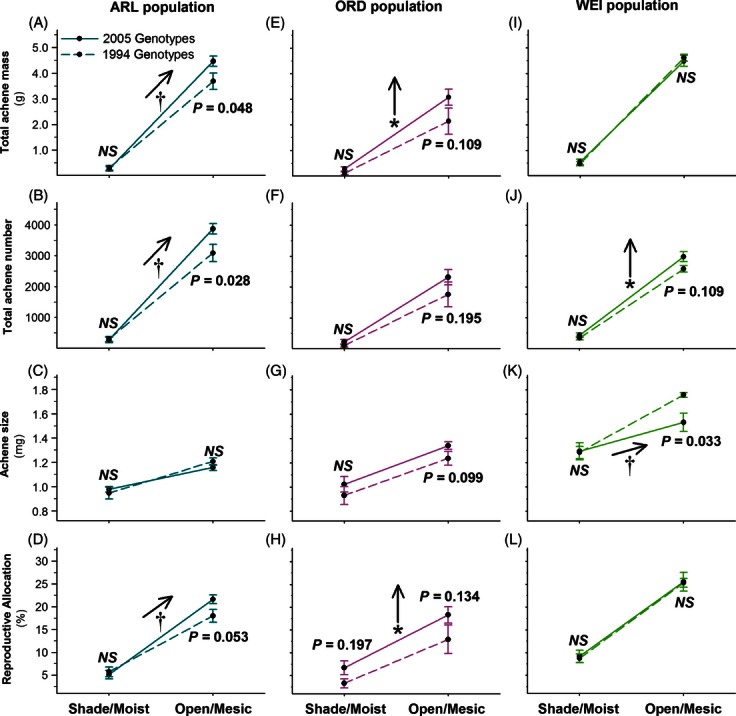
Population differences in fitness component evolution. Population means are shown ± 1 SE for 8–10 genotypes per collection year. Vertical arrows indicate significant main effect of Year in single-population anova; arrows parallel to 2005 norm of reaction indicate significant Treatment × Year effect. *P*-values are given from One-way anova testing for the effect of year within each population and habitat treatment. *P < 0.05; †*P* ≤ 0.10.

**Figure 3:**
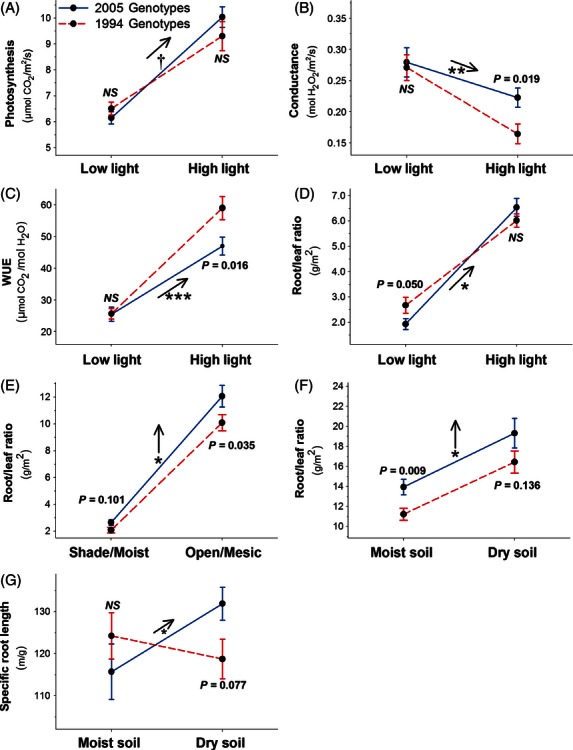
Rapid evolution of physiology and morphology. Means are shown ± 1 SE for 26–29 genotypes per collection year (pooled from three field populations; A–F) and for six genotypes per collection year (pooled from three field populations; G). Vertical arrows indicate significant main effect of year in full anova; arrows parallel to 2005 norm of reaction indicate significant Treatment × Year effect (see Tables 2 and 3). *P*-values are given from One-way anova testing for the effect of year within each treatment. ***P < 0.001; **P < 0.01; *P < 0.05; †*P* ≤ 0.10.

For each experiment, we calculated rates of evolutionary change in haldanes and Darwins for traits that showed significant year effects, i.e., for every trait in Figs 1–3 with a significant main or interaction effect of year (Hendry and Kinnison 1999). In cases where the main effect of year was significant, we calculated evolutionary rates for phenotypes expressed in both treatments in that experiment; in cases where the effect of year was significant only within a particular treatment, we calculated rates only for phenotypes expressed in that treatment.

## Results

Significant evolutionary change occurred in a number of fitness and functional traits between 1994 and 2005 (Tables 1–3, Year effects). Evolved changes in several traits were environment-specific (Tables 1–3, Treatment × Year effects), indicating evolution of plasticity. In some cases, evolutionary changes in mean trait values and/or plasticity differed among the three populations (Tables 1–3, Year × Population and Treatment × Year × Population effects).

**Table 1:** Effects of environmental treatment, collection year, and population on reproductive output in introduced *Polygonum cespitosum*

Experiment	Source of variation						

	Environmental treatment	Year	Population	Treatment × Year	Treatment × Population	Year × Population	Treatment × Year × Population
Habitat experiment	0.001***	0.008**	0.001***	0.085†	0.002**	0.781	0.604
*r*^*2*^ = 0.902; *N* = 93							
Light experiment	0.001***	0.001***	0.017*	0.769	0.732	0.844	0.634
*r*^*2*^ = 0.969; *N* = 71							
Moisture experiment	0.001***	0.062†	0.002**	0.471	0.959	0.076†	0.829
*r*^*2*^ = 0.912; *N* = 99							

*P*-values are shown from full anova (details in Materials and methods). Note that each row represents results of a separate experiment.

Statistical significance is indicated as: †*P* ≤ 0.10; *P < 0.05; **P < 0.01; ***P < 0.001.

**Table 2:** (a) Effects of light treatment (b) moisture treatment, collection year, and population on physiological traits in introduced *Polygonum cespitosum*

Physiological trait	Source of variation						

	Light treatment	Year	Population	Treatment × Year	Treatment × Population	Year × Population	Treatment × Year × Population
(a)							
Photosynthetic rate	0.001***	0.842	0.073†	0.051†	0.417	0.141	0.613
*r*^*2*^ = 0.724; *N* = 62							
Stomatal conductance	0.001***	0.778	0.363	0.005**	0.537	0.044*	0.672
*r*^*2*^ = 0.496; *N* = 62							
Water use efficiency	0.001***	0.241	0.956	0.001***	0.678	0.082†	0.944
*r*^*2*^ = 0.754; *N* = 62							

Physiological trait	Source of variation						

	Moisture treatment	Year	Population	Treatment × Year	Treatment × Population	Year × Population	Treatment × Year × Population
(b)							
Photosynthetic rate	0.001***	0.462	0.148	0.025*	0.616	0.039*	0.002**
*r*^*2*^ = 0.617; *N* = 53							
Stomatal conductance	0.036*	0.846	0.203	0.12	0.579	0.371	0.182
*r*^*2*^ = 0.288; *N* = 53							
Water use efficiency	0.001***	0.743	0.606	0.364	0.46	0.759	0.298
*r*^*2*^ = 0.495; *N* = 53							

*P*-values are shown from full anova (see Materials and methods).

Statistical significance is indicated as: †*P* ≤ 0.10; *P < 0.05; **P < 0.01; ***P < 0.001.

**Table 3:** Effects of environmental treatment, collection year, and population on root/leaf ratio (g/m^2^) in introduced *Polygonum cespitosum*

Experiment	Source of variation						

	Environmental Treatment	Year	Population	Treatment × Year	Treatment × Population	Year × Population	Treatment × Year × Population
Light Experiment	0.001***	0.705	0.856	0.039*	0.522	0.711	0.742
*r*^*2*^ = 0.643; *N* = 119							
Habitat Experiment	0.001***	0.014*	0.016*	0.149	0.255	0.195	0.642
*r*^*2*^ = 0.788; *N* = 97							
Moisture Experiment	0.001***	0.011*	0.575	0.928	0.896	0.171	0.947
*r*^*2*^ = 0.277; *N* = 108							

*P*-values are shown from full anova (details in Materials and methods). Note that each row represents results of a separate experiment.

Statistical significance is indicated as: †*P* ≤ 0.10; *P < 0.05; **P < 0.01; ***P < 0.001.

### Reproductive fitness

All three New England *P. cespitosum* populations have rapidly evolved to increase reproductive output in mesic or moist, high light conditions (Year and Year × Population effects, Table 1; Fig. 1A–C; note however that the Year effect was marginally non-significant for the Moisture Experiment). These increases reflect population-specific patterns of evolutionary change for particular components of fitness (Fig. 2; Population and Year × Population effects, Table S1). For instance, in the Habitat experiment, 2005 genotypes from the ARL population allocated more biomass to reproduction in the Open/Mesic treatment than those from 1994 (Fig. 2D), resulting in c. 25% greater total achene mass and number in this treatment (Fig. 2A,B). In the ORD population, 2005 genotypes increased reproductive allocation in *both* habitat treatments compared to 1994 plants (Fig. 2H) and also produced larger offspring in the Open/Mesic treatment (Fig. 2G), resulting in 30–35% greater achene mass and number across treatments (Fig. 2E,F). In contrast, 2005 genotypes from the WEI population did not increase reproductive allocation in either treatment compared to 1994 genotypes, and there was no change in total achene mass (Fig. 2L,I). However, because 2005 plants in this population *reduced* the size of their offspring in the Open/Mesic treatment compared to 1994 genotypes (though achenes remained significantly larger than those produced by other populations, *P* ≤ 0.05 based on Tukeys HSD test; Fig. 2K), they produced a greater number of offspring (Fig. 2J). Results of the Light and Moisture Experiments generally confirmed these population-specific evolutionary changes in reproductive allocation, offspring size, total achene mass and number (Supplement 1).

### Physiological response patterns

All three experimental populations evolved significantly different photosynthetic responses to High versus Low light and to Moist versus Dry soil between 1994 and 2005 **(**Year × Treatment effects, Table 2). Patterns of physiological plasticity in response to light evolved similarly in the three populations: in High light, 2005 genotypes increased photosynthesis more steeply (63% vs 43%) and decreased conductance less (20% vs 39%) than 1994 genotypes (Fig. 3A,B; cf. marginally significant Treatment × Year effect but NS Treatment × Population and 3-way interactions; Table 2). As a result of the shallower drop in conductance, 2005 genotypes increased water use efficiency (WUE) 18% less in High light than 1994 genotypes (Fig. 3C; Treatment × Year effect, Table 2).

By contrast, evolved changes in photosynthetic response to Dry vs. Moist soil differed dramatically among the three populations (Year × Population and Treatment × Year × Population effects, Table 2; Fig. S2D–F). In the ARL population, 2005 genotypes showed increased photosynthetic rates in both Dry and Moist soil compared to 2004 genotypes; 2005 ORD genotypes expressed significantly higher photosynthesis in Moist soil but *lower* photosynthesis in Dry soil, and WEI genotypes evolved to express lower photosynthetic rates across soil treatments (Fig. S2D–F). In contrast to the evolved changes in physiological responses to light, 2005 and 1994 genotypes from all three populations expressed similar stomatal conductance and WUE in response to Dry versus Moist soil (NS main and interaction effects of Year and Population, Table 2), with plants on average decreasing conductance by 18% and increasing WUE by 51% in Dry soil (significant Treatment effects, Table 2).

### Allocation and functional morphology

2005 genotypes expressed greater allocational plasticity in response to light level during the rapid growth stage (week 4) than 1994 plants, producing significantly less root tissue per unit of leaf surface area in Low light, and more steeply increasing this ratio in High light (mean within-genotype increase of 239% vs 125%, Fig. 3D; significant Treatment × Year effect, Table 3). 2005 genotypes also produced significantly more root mass per unit leaf area in response to the Shade/Moist and Open/Mesic habitat treatments, and to both Dry and Moist soils in full sun (Fig. 3E,F; significant Year effects, Habitat and Moisture experiments, Table 3). These evolutionary changes in allocational response were similar across the three populations (NS Year × Population and Treatment × Year × Population effects, all three experiments, Table 3), though populations differed on average in responses to the contrasting habitat treatments (Population effect, Table 3). Whole-plant root scans on a subsample of plants showed that 2005 and 1994 genotypes expressed markedly different plasticity patterns for root morphology in response to contrasting soil moisture levels (Fig. 3G). When grown in Dry versus Moist soil, 1994 genotypes decreased specific root length by 4%, while 2005 genotypes *increased* SRL in Dry soil by 14%. As a result, 2005 genotypes produced significantly longer roots per gram of tissue than 1994 genotypes in Dry soil (Treatment × Year effect *P* ≤ 0.075, *N* = 20; Fig. 3G; alpha significance level adjusted to 0.10 and population differences not tested in this small subsample).

### Calculated rates of evolutionary change

Rates of change calculated for the 14 cases of significant (main or interaction) effects of year across populations were low to moderate (Table 4A), ranging in absolute value from 0.0112 haldanes (= the NS change in achene number in the Shade/Moist treatment, Habitat Experiment; see Fig. 1A) to 0.1264 haldanes (= the significant change in SRL in the Dry treatment, Moisture Experiment; see Fig. 3G). Most rates of change (9 out of the 14 significant cases) fell between 0.038 and 0.078. Rates of change within single populations calculated for the 11 cases of significant (main or interaction) effects of year were generally similar (Table 4), ranging from 0.042 to 0.114 haldanes, with 9 of the 11 rate values falling between 0.06–0.09 haldanes (Table 4).

**Table 4:** Evolutionary rates are shown for traits with a significant Year effect in either the full anova or within-treatment 1-way anova for that trait (details in Materials and methods). In part (b), rates shown were calculated separately for each population based on results from the Habitat Experiment. Negative values indicate that the 2005 (ancestral) trait value is lower than the 1994 (descendant) trait value. Haldanes are expressed in phenotypic standard deviations per generation; Darwins are expressed in powers of e per million years (Hendry and Kinnison 1999)

Experiment	Trait	Treatment	Haldanes	Darwins (×10^−3^)	Figure
(a) Evolutionary rates in introduced *Polygonum cespitosum*					
Habitat experiment	Achene number	Open/Mesic	0.0437	13.67	1A
Habitat experiment	Achene number	Shade/Moist	0.0112	8.48	1A
Light experiment	Achene number	High Light	0.0707	15.38	1B
Light experiment	Achene number	Low Light	0.0775	29.79	1B
Moisture experiment	Achene number	Dry Soil	0.0196	4.44	1C
Moisture experiment	Achene number	Moist Soil	0.0500	12.21	1C
Light experiment	Conductance	High Light	0.1008	27.63	3B
Light experiment	WUE	High Light	−0.1040	−20.69	3C
Light experiment	Root/leaf ratio	Low Light	−0.0499	−29.50	3D
Habitat experiment	Root/leaf ratio	Open/Mesic	0.0537	17.31	3E
Habitat experiment	Root/leaf ratio	Shade/Moist	0.0453	19.07	3E
Moisture experiment	Root/leaf ratio	Dry Soil	0.0376	14.69	3F
Moisture experiment	Root/leaf ratio	Moist Soil	0.0677	19.69	3F
Moisture experiment	SRL	Dry Soil	0.1264	9.54	3G

Trait	Treatment	Population	Haldanes	Darwins (×10^−3^)	Figure
(b) Within-population evolutionary rates in introduced *Polygonum cespitosum*					
Achene mass	Open/Mesic	ARL	0.0841	17.53	2A
Achene number	Open/Mesic	ARL	0.1036	20.54	2B
Allocation	Open/Mesic	ARL	0.0867	16.80	2D
Achene mass	Open/Mesic	ORD	0.0821	32.87	2E
Achene mass	Shade/Moist	ORD	0.0635	85.86	2E
Achene number	Open/Mesic	ORD	0.0617	24.98	2F
Achene size	Open/Mesic	ORD	0.0881	7.34	2G
Allocation	Open/Mesic	ORD	0.0797	31.68	2H
Allocation	Shade/Moist	ORD	0.0734	64.67	2H
Achene number	Open/Mesic	WEI	0.0848	12.75	2J
Achene number	Shade/Moist	WEI	0.0424	20.99	2J
Achene size	Open/Mesic	WEI	−0.1138	−12.52	2K

## Discussion

In this study, we used a newly invasive introduced species to investigate a fundamental evolutionary issue: the potential for rapid evolution of ecologically important traits, including adaptive plasticity. The results may be relevant to other introduced taxa; more generally, they illuminate the timely and indeed urgent issue of potential evolutionary response to novel environmental conditions. Rates of evolutionary change in introduced-range populations of *P. cespitosum* calculated in haldanes (phenotypic standard deviations per generation, the most appropriate measure for microevolutionary studies, Hendry and Kinnison 1999) were similar to those for evolution of tolerance to heavy metals and road de-icing salt in herbaceous plant populations, but less than the very high rates of evolution that have been documented in several plant taxa for growth traits in response to herbicides (reviewed by Bone and Farres 2001). With respect to species introductions, rates of change in the *P. cespitosum* populations were higher than rates of evolution for growth traits in introduced versus native populations of several biennial and perennial herbs, but lower than the dramatic rates documented for evolution of herbivore resistance in the highly invasive beachgrass *Spartina* (Bone and Farres 2001). Evolutionary rates calculated in the present study also fell within the range for published studies of contemporary evolution in molluscs, fish and reptiles (reviewed by Hendry and Kinnison 1999).

### Rapid evolution of reproductive success in open conditions

Comparisons of 1994 and 2005 genotypes from three *P. cespitosum* populations reveal that the species has rapidly evolved in its introduced range to increase reproductive fitness in full sun, very moist to mesic conditions. Alternative explanations for this specific change across years are very unlikely to apply. First, gene flow is quite limited in this gravity-dispersed species, so it is very unlikely that these within-population changes are due to gene flow from hypothetical sun-adapted populations nearby (this is even more unlikely to have occurred in all three geographically distinct populations). Furthermore, trait phenotypes expressed in shade/moist (i.e., ancestral) conditions were in many cases identical to those produced by the 1994 genotypes of the same population, which would not be the case if the populations had been recolonized from other sites. Finally, the populations are clearly genetically distinct from one another, which would not be the case if they had all received immigrants from a common, differently-adapted source. (Both limited gene flow and substantial among-population genetic diversity have been confirmed in a related microsatellite study; S. Matesanz, K. Theiss, K. Holsinger and S. Sultan, unpublished data).

This experimental result is strikingly consistent with field data indicating that in the past 1–2 decades, *P. cespitosum* has begun to colonize open, moist to dry conditions, in addition to the shaded sites that characterize both its native distribution and its initial distribution in NE North America (Fig. S1). Greater fitness in full sun appears not to entail a trade-off with performance in shade: in all three populations, 2005 genotypes maintained or slightly increased reproductive output in shaded treatments relative to 1994 plants. In its introduced range, *P. cespitosum* is thus evolving genotypes able to succeed in a broader range of environmental conditions. In contrast to the ecological specialization that results from fitness trade-offs, the ability to both maintain fitness in resource-limited (e.g., low light) conditions and opportunistically maximize reproduction in resource-rich (e.g., full sun) environments is a pattern of fitness plasticity predicted to characterize generalist and invasive taxa (Sultan 2001; Alpert and Simms 2002; Richards et al. 2006).

### Rapid evolution of functional trait plasticity

Populations in the introduced range have also recently evolved appropriate functional responses to high versus low light and to dry soil. As a result of changes in both photosynthesis and stomatal conductance, 2005 genotypes had higher rates of carbon fixation in moist, full sun conditions, and significantly lower water use efficiency, than those from 1994. High photosynthetic rate with low WUE is considered a favorable growth strategy for annual plants in moist conditions (Arntz and Delph 2001; Geber and Griffen 2003), since reduced WUE associated with high conductance adversely affects fitness only in dry soils (Heschel et al. 2004; Saldaña et al. 2007). Introduced *P. cespitosum* genotypes from 1994 were previously found to have high WUE in full sun relative to annual congeners due to comparatively low rates of both photosynthesis and conductance (Sultan et al. 1998a). The evolution of higher metabolic rates in full sun coincides with the species' recent transition from a shade-restricted herb to one inhabiting a wider range of light environments, supporting the view that evolution of novel, adaptive expression patterns for functional traits can promote colonization of new habitats (Lee et al. 2003; Richards et al. 2006).

Along with these changed physiological responses, plants in the study populations have rapidly evolved altered plasticity patterns for root allocation and morphology. The greater root-to-leaf allocation of 2005 plants in high light conditions would maximize the supply of water and nutrients to photosynthetic tissues, allowing opportunistically high rates of carbon assimilation despite the transpirational demands of open habitats (Lechowicz and Blais 1988; Fitter and Hay 2002). This plastic response was even more pronounced in 2005 plants growing in mesic and dry soil in full sun, consistent with the increased root uptake surface needed to access limited soil moisture. *P. cespitosum* populations have also evolved substantially modified, functionally adaptive plasticity for root morphology (Bell and Sultan 1999), substantially increasing the root length (and hence absorptive surface area) per gram of root tissue in dry, full-sun conditions.

Certain evolved changes in allocational plasticity are also consistent with adaptive ecophysiological predictions for shade phenotypes. Relative to 1994 genotypes, 2005 plants decreased root-to-leaf ratio in moist shade (Light Experiment, week 4), where growth depends on maximizing photosynthetic surface area rather than roots (Fitter and Hay 2002). However, this changed response appears to be expressed only in juvenile plants, since it was not found in plants grown in the Shade/Moist habitat treatment that were harvested at senescence. A recent meta-analysis comparing populations before and after anthropogenic disturbance found that evolved changes in population-level ‘plasticity’ (defined as change in the population's response slope, in the absence of environment-specific adaptive criteria) were idiosyncratic in plants, with no clear trends in the direction of change for specific types of trait (Crispo et al. 2010). The changes here documented in *P. cespitosum* also include both increases and decreases in slope (for instance, the evolution of a shallower decrease in conduction rates in high light, corresponding to steeper increases in photosynthetic rates and fitness in that treatment).

### The selective context for rapid adaptive evolution in *P. cespitosum*

Because light and moisture conditions at the study populations have not altered (see Materials and methods, Experimental sample), it seems unlikely that these rapid adaptive changes reflect a directional selective response to increased light. Rather, these changes in physiological, developmental and fitness plasticity may result from selection in the patchy, temporally-variable light milieu of disturbed shade habitats in the species' introduced range (microsite data in T. Horgan-Kobelski, S. Matesanz, & S. E. Sultan, unpublished manuscript). Pre-existing plasticity may have allowed introduced genotypes of *P. cespitosum* to colonize these heterogeneous sites, providing a starting point for further adaptive evolution (West- Eberhard 2003; Ghalambor et al. 2007; Moczek et al. 2011; see also Levin 2009). Because effectively harvesting intense light sharply increases reproductive output, subsequent selection in these variable habitats would strongly favor genotypes able to functionally exploit high light conditions, even if fully open microsites were comparatively rare or intense insolation only intermittent (Kawecki et al. 1997). Selection within these patchy habitats could thus have led to the evolution of adaptive plastic responses to high light (and concomitant moisture deficits). Furthermore, such functional plasticity would have pre-adapted *P. cespitosum* genotypes to colonize open sites (see Donohue et al. 2001), possibly facilitating the species' ecological range expansion into sunnier and potentially drier habitats.

The lack of detailed environmental information regarding *P. cespitosum'*s ecological distribution in its Asian home range makes it difficult to assess the species' apparent failure to evolve adaptations allowing its spread into sunny habitats during its long evolutionary history in that range. Possibly the more uniform shade conditions of undisturbed Asian forest understories provided no selective advantage to genotypes able to express high light-adapted phenotypes. It is also possible that open habitats in the Asian range include abiotic or biotic stresses beyond the tolerance of *P. cespitosum* genotypes, such as specialized herbivores, pathogens, or competitors, or extreme and/or prolonged moisture deficits. Future studies characterizing the species' ecological range in Asia would help to resolve this question.

### Implications for invasion biology

To our knowledge, this is the first study to directly document the evolution of increased plasticity for key functional traits in the introduced range of an invasive species. While both adaptive evolution and phenotypic plasticity have been considered important drivers of species invasions (Sakai et al. 2001; Sultan 2004; Richards et al. 2006), they have often been viewed as separate factors; few studies have examined adaptive evolution of plasticity in introduced species. Nonetheless, such evolution may be a common, critically important event during the well-known lag time between an invasive species' initial introduction to a new range and its later, often sudden switch to aggressive spread (Sakai et al. 2001; Lee 2002). Several studies have found greater plasticity of invasive species in their introduced than in their native ranges (reviewed in Bossdorf et al. 2005; Matesanz et al. 2010), but these studies could not distinguish colonization by a subset of highly plastic, pre-adapted genotypes (e.g., Bossdorf et al. 2008) from evolution of plasticity in the introduced range. Neutral molecular evidence indicates that in some cases (e.g., Lavergne and Molofsky 2007) increased plasticity may have evolved within a taxon's introduced range, as has evidently occurred in *P. cespitosum*. Further resurrection studies of introduced plants, animals, and microorganisms will help to clarify whether rapid evolution of increased plasticity is a common feature of invasive species. More generally, our results suggest that resurrection approaches could be of broad utility in conservation biology, both to identify recent evolutionary changes that can contribute to invasiveness, and to test for either adaptive or maladaptive contemporary evolution in populations of rare or threatened taxa.

### Evolutionary potential in populations of invasive species

Evolution of functional and fitness responses has occurred within all three study populations of *P. cespitosum* over a recent 11-generation period, demonstrating remarkably rapid adaptive change in an introduced species. In this instance, evolution of responses to full-sun conditions is consistent with the concomitant spread of the species from its original shade distribution into open habitats, and its new status as an invasive in the region.

Along with this shared pattern of adaptive change, differences persist among the populations in absolute reproductive output in both open and shade conditions (cf. 2005 genotypes, Fig. 2A, E, I). These population differences in fitness provide an additional, equally fundamental insight to evolutionary change in introduced species. They suggest that idiosyncratic genetic constraints and/or local selective pressures may differently limit adaptive change in populations of introduced taxa (Lee 2002), as is the case in native species exposed to novel environmental challenges (e.g., Al-Hiyaly et al. 1988). Indeed, such population differences can be expected as a result of founder effects and random genetic drift in self-fertilizing species with limited dispersal such as *P. cespitosum*. As a consequence, distinct populations may possess different potentials to persist in, and further adapt to, new habitats within an introduced range. Accordingly, the expanded ecological repertoire that can fuel a species' invasion in a new region may result from evolutionary change in only a subset of its populations (Sakai et al. 2001; Lee and Gelembiuk 2008), suggesting that invasion dynamics may be shaped in part by population-level evolutionary events and constraints.

## Data Archiving Statement

Data underlying the main results of this study are available as online supplemental materials.

## Supporting Information

**Figure S1** Recent ecological expansion of *Polygonum cespitosum* in its introduced range.

**Figure S2** Among-population trends in morphological and physiological evolution.

**Table S1** Effects of habitat treatment, collection year, and population on fitness components in introduced *Polygonum cespitosum*.
