# Supplementary material for: A resurrection study reveals rapid adaptive evolution within populations of an invasive plant
Source: Evol Appl. 2012 Sep 9;6(2):266–78. doi: 10.1111/j.1752-4571.2012.00287.x (PMC3689352; doi:10.1111/j.1752-4571.2012.00287.x)
Supplement: Supplementary file 1 [file eva0006-0266-SD1.jpg]

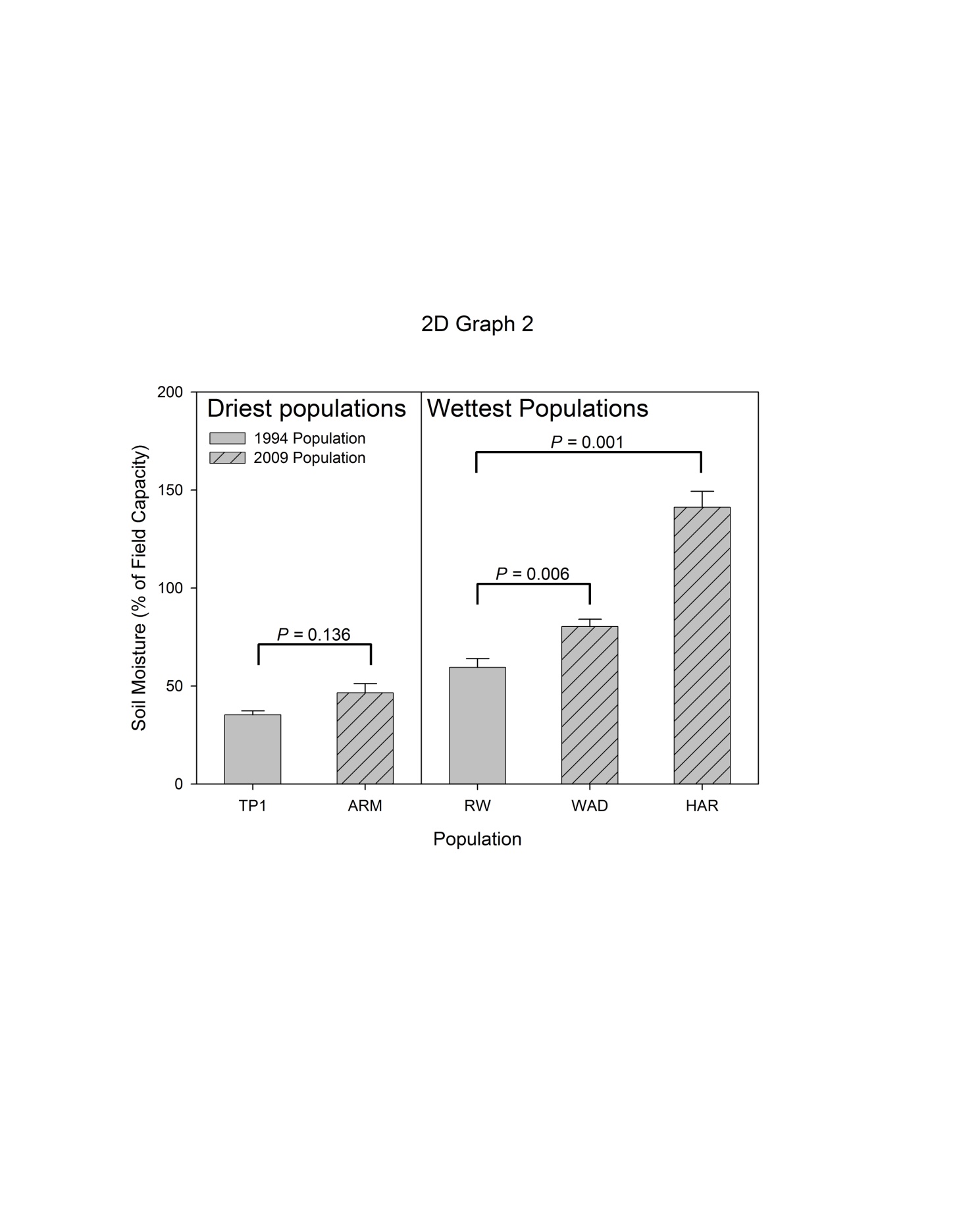

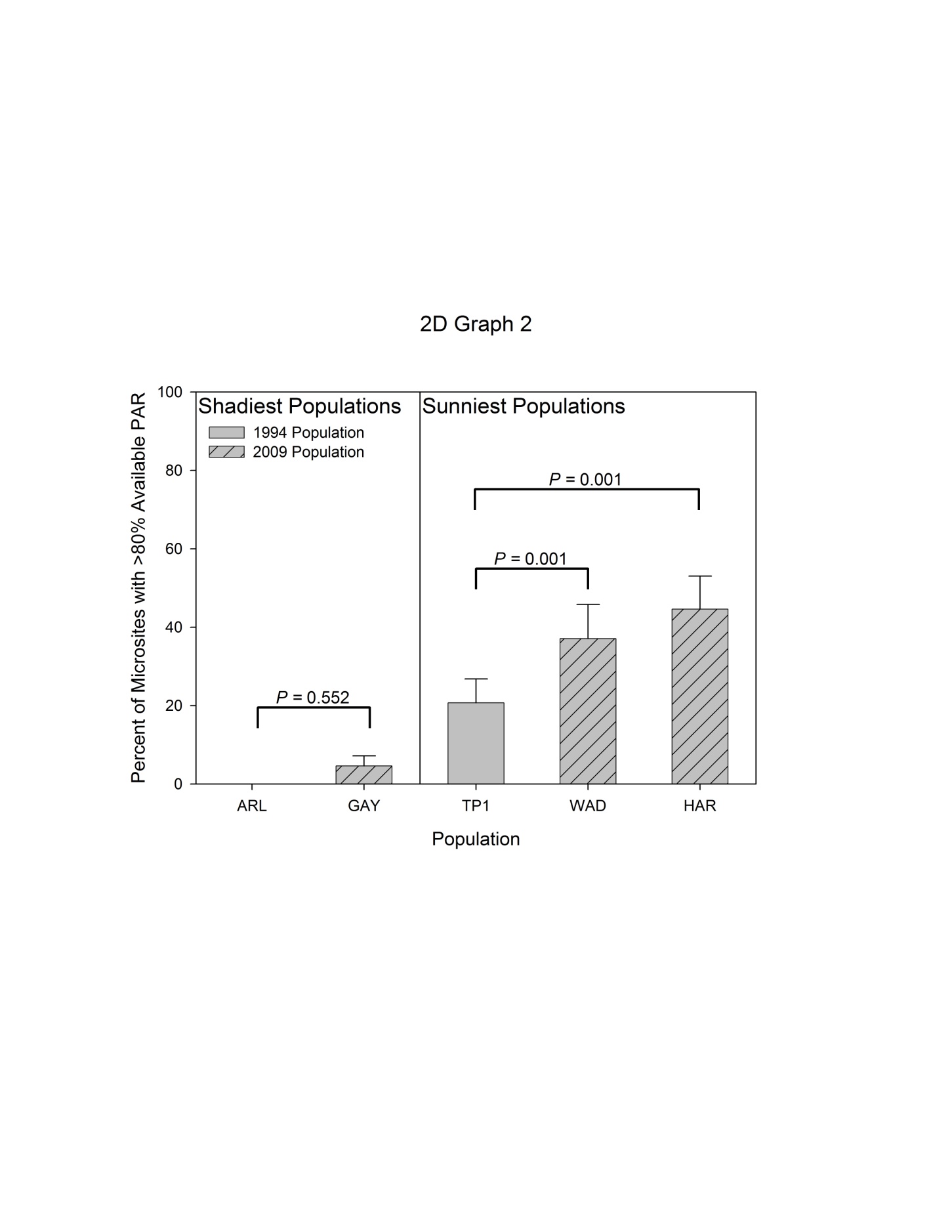


**a b**

**Figure S1: Recent Ecological Expansion of *Polygonum cespitosum* in its Introduced Range.** Site means ± 1SE for (a) soil moisture and (b) insolation, reported as the proportion of microsites receiving >80% of available Photosynthetically Active Radiation (PAR) for *P. cespitosum* populations in northeastern North America. The driest, wettest, shadiest, and sunniest population sites are shown from a set of 18 populations (1994) and 14 populations (2009) that were initially sampled (complete data in Sultan *et al.* 1998 and Horgan-Kobelski *et al.*, *unpublished ms*.). Soil moisture was determined based on samples collected from two depths (0-10cm and 20-30cm) at each of 8-10 microsites per population site at two timepoints during *P. cespitosum*’s growth season (N= 32-40 samples per site). Moisture content was converted to a percentage of the soil’s field capacity (a proxy for soil water potential), as determined for each site and depth. The proportion of microsites at each site receiving >80% of available PAR was calculated from a sample of 15-16 0.8-meter transects (each with 8 quantum sensors spaced 10 cm apart; Accupar LP-80 sunfleck ceptometer, Accupar Inc.) for each site, taken on sunny days at both *Polygonum* canopy and mid-canopy levels at two timepoints during the growth season (N=60-64 transects per site).

To test for ecological range expansion, the most extreme population site (*i.e.* the wettest site, the driest site, the shadiest site, and the sunniest site) in 1994 was compared to the most extreme sites in 2009. *P* values are shown from linear contrasts following a mixed-model ANOVA testing for the effects of Year, Population (nested within Year) and Season (early vs. late) on each variable; models included the effect of Height (canopy vs. mid-canopy) on light and Depth (0-10cm vs. 20-30cm) on soil moisture (details in Horgan-Kobelski *et al.*, *unpublished ms*). In cases where the contrast showed a significant change between the extreme 1994 and 2009 sites for a given environmental factor, the second most extreme site from the year with the broader range was compared to the most extreme site from the year with the narrower range to verify that the measured change in ecological range was not due to sampling an extremely unusual site by chance (van Valen 2005). Contrasts show that during the sampling interval, *P. cespitosum* populations in this region have expanded into both wetter and sunnier sites. Both the wettest (HAR) and second wettest (WAD) 2009 sites had significantly higher mean soil moisture than the wettest site from 1994 (RW), while the driest 1994 site (TP1) and the driest 2009 site (ARM) did not differ significantly in soil moisture. Both the sunniest (HAR) and second sunniest (WAD) sites in 2009 had significantly higher proportions of high-light microsites than the sunniest site from 1994 (TPW), while the shadiest sites from 1994 (ARL) and 2009 (GAY) did not differ significantly in the proportion of high-light microsites. Note that the driest 2009 site (ARM) also had high average light intensity, due to a moderate proportion of fully open (ie, high light/mesic to dry) microsites (data in Horgan-Kobelski *et al.*, *unpublished ms*.).
