# Supplementary figures and images for: A resurrection study reveals rapid adaptive evolution within populations of an invasive plant
Source: Evol Appl. 2012 Sep 9;6(2):266–78. doi: 10.1111/j.1752-4571.2012.00287.x (PMC3689352; doi:10.1111/j.1752-4571.2012.00287.x)

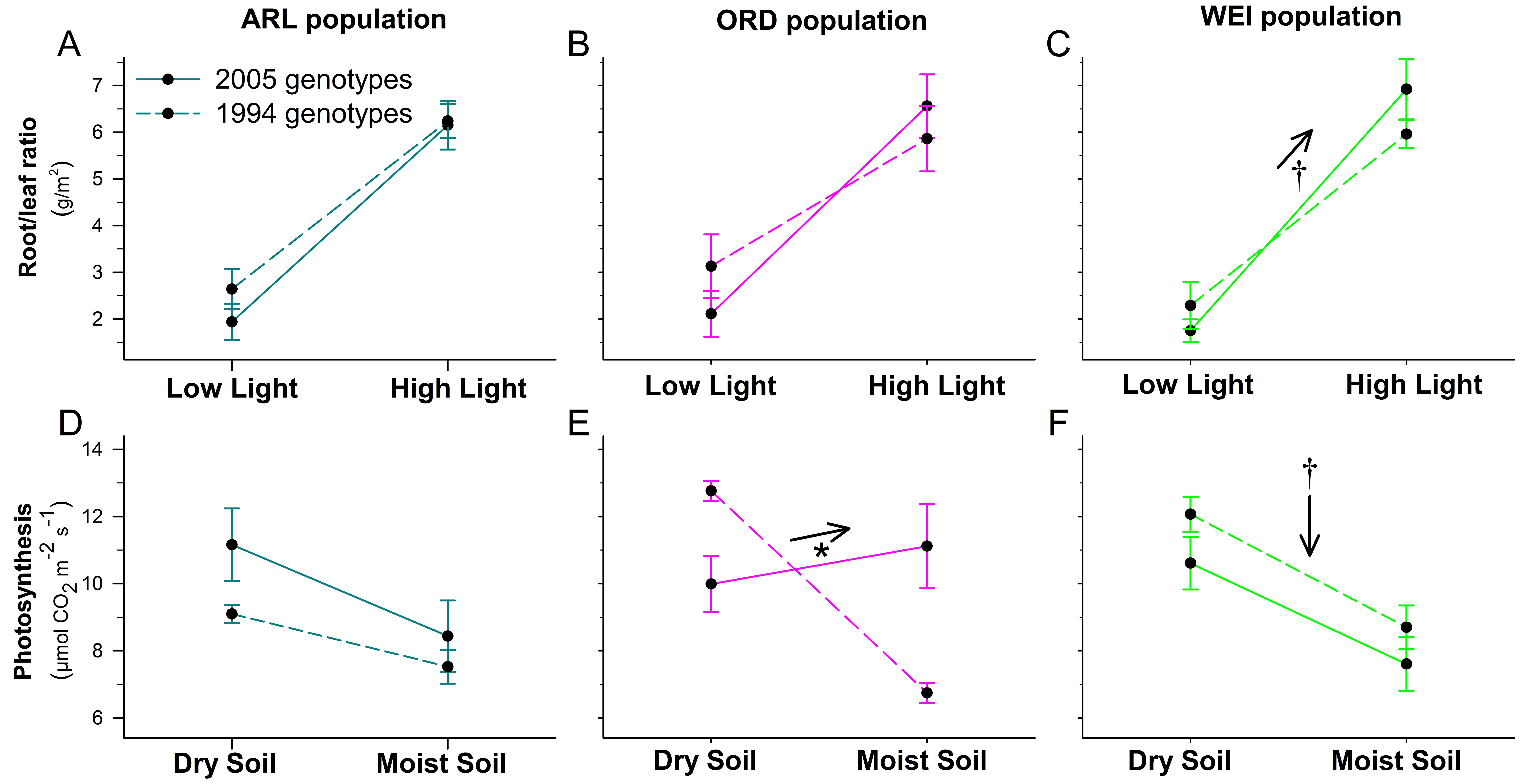

Supplement: Supplementary file 2 [file eva0006-0266-SD2.docx]
