# Supplementary material for: A resurrection study reveals rapid adaptive evolution within populations of an invasive plant
Source: Evol Appl. 2012 Sep 9;6(2):266–78. doi: 10.1111/j.1752-4571.2012.00287.x (PMC3689352; doi:10.1111/j.1752-4571.2012.00287.x)
Supplement: Supplementary file 3 [file eva0006-0266-SD3.xlsx]

| **Table S1: Effects of habitat treatment, collection year, and population on fitness components in introduced *Polygonum cespitosum*** | | | | | | | | |
| --- | --- | --- | --- | --- | --- | --- | --- | --- |
|  | Source of variation | | |  |  |  |  |  |
| **Fitness component** | Habitat Treatment | Year | Population | Treatment x Year | Treatment x Population | Year x Population | Treatment x Year x Population | |
|  |  |  |  |  |  |  |  |  |
| **Total achene mass** | **0.001***** | **0.012*** | **0.001***** | 0.292 | **0.008**** | 0.256 | 0.164 |  |
| *r^2^* = 0.912; N = 99 |  |  |  |  |  |  |  |  |
|  |  |  |  |  |  |  |  |  |
| **Mean individual achene mass** | **0.001***** | 0.826 | **0.001***** | **0.102†** | 0.157 | **0.032*** | 0.264 |  |
| *r^2^* = 0.739; N = 93 |  |  |  |  |  |  |  |  |
|  |  |  |  |  |  |  |  |  |
| **Allocation to reproduction** | **0.001***** | **0.024*** | **0.001***** | 0.274 | **0.043*** | 0.208 | 0.562 |  |
| *r^2^* = 0.782; N = 97 |  |  |  |  |  |  |  |  |
|  |  |  |  |  |  |  |  |  |
| *P*-values are shown from full ANOVA (see Materials and methods). | | | |  |  |  |  |  |
| Statistical significance is indicated as: † P ≤ 0.10; * P < 0.05; ** P < 0.01; *** P < 0.001. | | | | | | |  |  |
