# Supplementary material for: A resurrection study reveals rapid adaptive evolution within populations of an invasive plant
Source: Evol Appl. 2012 Sep 9;6(2):266–78. doi: 10.1111/j.1752-4571.2012.00287.x (PMC3689352; doi:10.1111/j.1752-4571.2012.00287.x)
Supplement: Supplementary file 4 [file eva0006-0266-SD4.docx]

***Supplement 1***

***Results of fitness component evolution, Light and Moisture Gradients***

Genotypes from ARL evolved increased allocation to reproduction in the Moisture Gradient (effect of **Year** *P*=0.055 in single-population ANOVA; reproductive allocation data were not collection in the Light Gradient), and increased achene mass and number in both the Light and Moisture gradients (effect of **Year** *P* ≤ 0.02 for both traits in single-population ANOVA, both experiments). Genotypes from ORD evolved increased achene mass and number in the Light Gradient (effect of **Year** *P* ≤ 0.02 for both traits in single-population ANOVA) and non-significantly increased total achene mass in the Moist treatment of the Moisture Gradient (*P*=0.111 in one-way ANOVA). In contrast to these increases, genotypes from WEI showed no evidence of fitness component evolution in the Moisture Gradient. As in the habitat experiment, WEI 2005 genotypes in the Light Gradient reduced individual achene mass by 14% across light treatments compared with 1994 genotypes (effect of **Year** *P*=0.031 in single-population ANOVA), though WEI 2005 genotypes also produced fewer achenes in the High Light treatment than did 1994 genotypes (Fig. 1b; significant **Year x Population** effect, Table 1). This anomalous reduction in reproductive output is likely due to the early-achene harvesting procedure used in this experiment (see *Materials and methods*), which resulted in an under-estimate of total reproductive output of 2005 WEI genotypes due to their later phenology.
